# Preoperative Challenges in Managing Intraparotid Schwannoma

**DOI:** 10.7759/cureus.21392

**Published:** 2022-01-18

**Authors:** Mohamed Iliyas Sultan Abdul Kader, Asma Abdullah, Mohd Razif Mohamad Yunus, Mohd Najib Jaafar, Thean Yean Kew

**Affiliations:** 1 Department of Otorhinolaryngology – Head and Neck Surgery, Universiti Kebangsaan Malaysia Medical Centre, Kuala Lumpur, MYS; 2 Department of Otorhinolaryngology – Head and Neck Surgery, Hospital Melaka, Melaka, MYS; 3 Department of Radiology, Universiti Kebangsaan Malaysia Medical Centre, Kuala Lumpur, MYS

**Keywords:** schwannoma, parotid, imaging, facial nerve, head and neck neoplasms

## Abstract

Schwannomas are a benign and rare entity that originates from Schwann cells. The majority of schwannomas are found in the head and neck regions and usually involve the intratemporal course of the facial nerve (FN). Isolated extratemporal intraparotid involvement is very rare. It is very challenging to diagnose intraparotid facial nerve schwannoma (PFNS) based on fine needle aspiration cytology (FNAC) preoperatively. We report a case of an intraparotid facial nerve schwannoma masquerading as pleomorphic adenoma. The diagnostic challenges and imaging features along with its management are discussed.

## Introduction

Schwannomas are benign and rare neuroectodermal tumors arising from Schwann cells of nerve sheaths. They are also known as neurilemmomas and neurinomas [1]. Schwannomas involve the head and neck region approximately 25%-40% of the time. Most of the facial nerve schwannomas arise from the intratemporal part of the facial nerve (FN). Extratemporal involvement is only 9% [2]. There are only 83 patients with isolated parotid involvement reported worldwide [3]. Intraparotid facial nerve schwannomas (PFNS) usually present as a slow-growing painless mass with normal facial function [3].

## Case presentation

**Figure 1:**
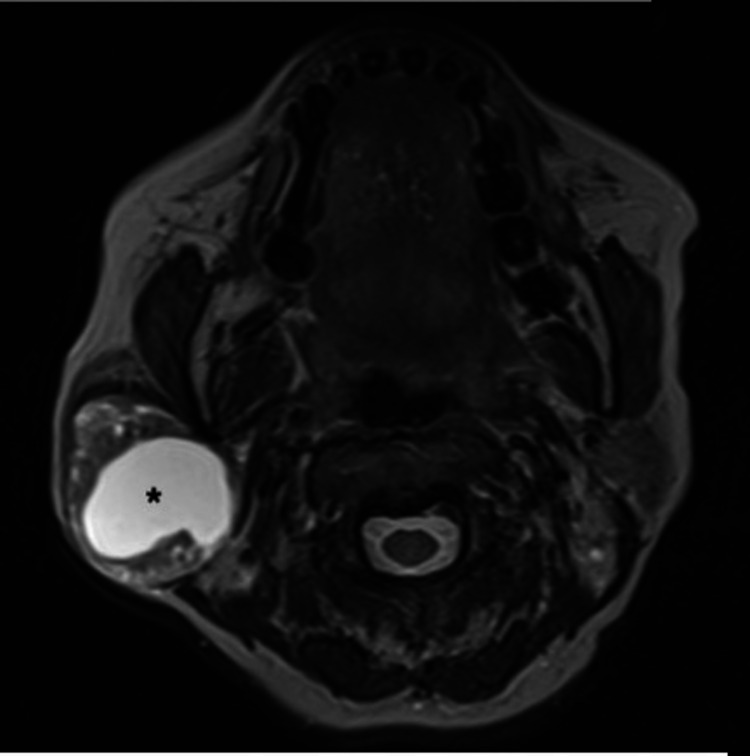
T2-weighted MRI (T2WI). Image showing a well-defined lesion in the right parotid gland with a central homogeneous hyperintense component (*) suggestive of a cyst. Note the heterogeneous peripheral solid component with a few smaller microcysts within.

**Figure 2:**
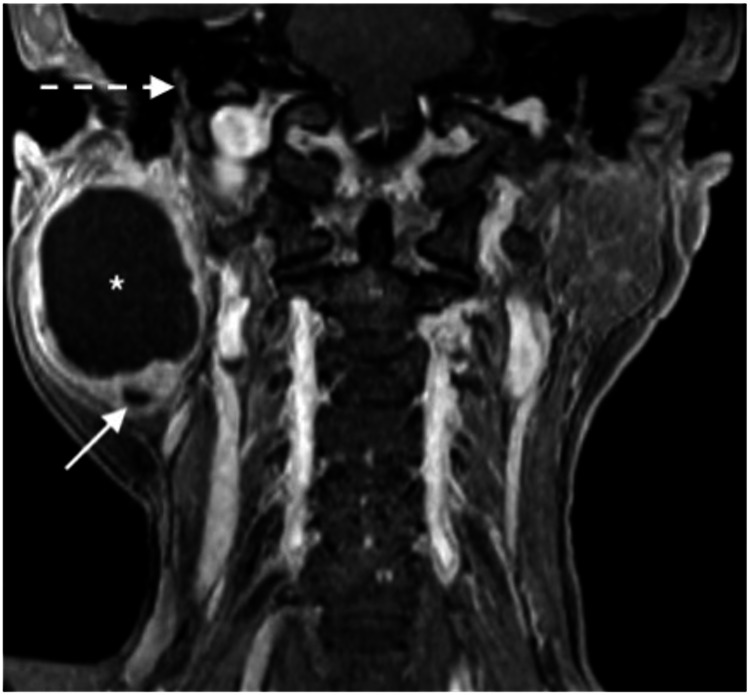
T1 VIBE fat-saturated image. In a post-gadolinium image, the peripheral solid component enhances, surrounding the central non-enhancing hypointense cystic component (*). A smaller cyst (arrow) is also noted within the peripheral solid component. Note the normal mastoid segment of the right facial canal (dotted arrow) in relation to the parotid lesion.

A 60-year-old female presented with a painless right infra-auricular swelling for 20 years. The mass was not increasing in size, and she does not have any facial asymmetry. On examination, there was a right parotid swelling measuring about 4 × 4 cm. FN examination showed House-Brackmann (HB) grade I, which was normal. Other cranial nerves, ear, neck, and nasopharynx examinations were unremarkable. Final needle aspiration cytology (FNAC) of the right parotid mass showed mixed myoepithelial and stromal cells reported as pleomorphic adenoma. The T2-weighted image (T2WI) showed a well-defined lesion in the right parotid gland with a central homogeneous hyperintense component suggestive of a cyst (Figure 1), and on the coronal reconstructed image from T1 VIBE fat-saturated image (T1 VIBE FS), a peripheral solid component surrounds the central non-enhancing hypointense cystic component; a smaller cyst was also noted within the peripheral solid component (Figure 2).

The patient underwent right superficial parotidectomy under general anesthesia. The FN was identified anatomically and physiologically. However, the lower division of the FN is blended with the tumor. Upon tracing the FN, it involves the superior and medial parts of the tumor. Postoperation, she developed grade V FN palsy. Postoperative histopathological examinations confirmed it to be schwannoma, which showed circumscribed lesion composed of spindle cell proliferation arranged predominantly in cellular Antoni A pattern. The spindle cells had oval, tapered nuclei with poorly defined eosinophilic cytoplasm. Verocay body formation and hyalinized blood vessels are seen. Immunohistochemistry showed nuclear and cytoplasmic reactivity for S100.

Postoperatively, the patient underwent regular facial physiotherapy. At six-month follow-up, her FN function improved to HB grade II.

## Discussion

Schwannomas can be sporadic or often associated with neurofibromatosis type 2, Von Recklinghausen’s disease, and schwannomatosis and common in postradiation patients [4]. The most affected cranial nerves are vestibular, trigeminal, and hypoglossal nerves [5]. Facial nerve involvement is rare, but when it occurs, the tumor can originate at any part of the nerve, from the cerebellopontine angle to its peripheral branches. The intratemporal segment is the commonest site of development (71%), whereas intraparotid represents only 9% [2,4].

FN paresis, paralysis, and twitching were more commonly seen in intratemporal FN schwannomas in up to 61% of cases, while only 18.5% of patients with PFNS along with intratemporal extension had FN symptoms [3]. However, only 0.04% of patients with exclusive PFNS have FN symptoms. Commonly, PFNS presents as a painless parotid mass. Other symptoms include numbness, discomfort, local irritation, twitching, and auricular symptoms [6,7].

All these factors make PFNS very challenging and difficult to diagnose preoperatively. In cases of PFNS with intratemporal extension, a CT scan will reveal a smooth, well-circumscribed intraparotid lesion along with dilation of the fallopian canal. MRI remains the gold standard investigation of choice for assessing detailed FN anatomy [3,8]. Gadolinium-enhanced MRI will demonstrate an isointense to hypointense T1 and isointense to hyperintense T2 signal with avid contrast uptake [8]. There are no definitive radiological findings for PFNS.

Parotid cystic lesions can generally be categorized into three groups: nonneoplastic cysts, benign tumors with macrocystic change [9], and malignant tumors with macrocystic change. The preoperative diagnosis of these cystic lesions is crucial as it affects the patient’s management.

Nonneoplastic cysts are typically seen on MRI as cystic lesions with a well-defined margin and no solid component. However, there are few reported cases of benign tumors with extensive cystic degeneration, which show the presence of mural nodules within the cystic component [10].

As for our case, the solid cystic appearance and presence of enhanced solid component are indicative of a tumor and exclude the possibility of nonneoplastic cysts. The parotid lesion also shows a well-defined margin on gadolinium-enhanced MRI, thus making a malignant tumor less likely.

Radiologically, the differential diagnosis of benign parotid tumor for this patient includes Warthin’s tumor, pleomorphic adenoma, and PFNS. Warthin tumor is favorable at the time of reporting rather than pleomorphic adenoma as cystic degeneration is more commonly seen in the former. The diagnosis of PFNS is challenging in our case, as there are no target and string signs demonstrated, although the parotid lesion is situated below the skull base and posterolateral to the retromandibular vein (Table 1) [2].

**Table 1:** Summary of the main differential diagnosis for solid cystic lesion of the parotid gland in our case.

Benign parotid tumor	MRI description
Warthin’s tumor	Solid component
	T2WI: isointensity to hypointensity
	ADC value: low
	DCE: washout pattern
	Cyst: T1WI hyperintensity
Pleomorphic adenoma	Lobulated contour, complete capsule
	Solid component
	T2WI hyperintensity
	ADC value: high
PFNS	Target sign on T2WI
	Growth toward the facial canal
	String sign

A retrospective study done by Caughey et al. consisting of 3722 patients with schwannomas over a 38-year period reveals only 29 cases associated with FN. Among these 29 cases of facial nerve schwannomas, only eight patients had intraparotid involvement [11].

The MRI features of PFNS that have been described in the literature include tumor growth toward the facial canal, target sign, and string sign [2,12]. The characteristic location of extratemporal schwannomas is posterolateral to the retromandibular vein with extension toward the stylomastoid foramen of the facial canal [13]. The appearance of peripheral hyperintensity and central hypointensity on T2WI is the definition of the target sign. In schwannoma, this is due to the corresponding loose myxomatous Antoni B region in the periphery and densely cellular Antoni A region in the center seen microscopically [14]. However, the target sign is not specific as it is also seen in plexiform neurofibromas [14]. Jaiswal et al. described a string sign characterized by the presence of the parotid mass below the stylomastoid foramen with beaking into the foramen [12].

Martin et al. reported PFNS as slightly heterogeneous lesions with isointense signal intensity on T1WI and T2WI [15], whereas Shimizu et al. described PFNS showing an isointense signal to muscle on T1WI and hyperintense signal to muscle on T2WI [2]. In our study, the peripheral solid component is isointense to the muscle on T1WI and T2WI, while cystic components demonstrate hypointensity on T1WI and hyperintensity on T2WI.

Due to its rarity, there is no worldwide consensus on the management of PFNS. Gross et al. suggested the management of PFNS according to tumor location, its morphology with regard to FN anatomy, and preoperative FN function to dictate the treatment plan, focusing on long-term neurologic preservation [3].

Gross et al. suggested en bloc tumor nerve resection with interposition nerve graft for tumors that are 1) loosely attached with small terminal midface branch and 2) inseparable with preoperative HB IV-VI FN function. However, in patients with good preoperative FN function (HB I-III), watchful waiting with serial imaging or examinations should be offered. PFNS are slow-growing tumors with low malignant potential. Hence, the principle of treatment is to preserve FN function, cosmesis, and long-term tumor control [3].

In our patient, to improve the accuracy of the diagnosis, FNAC or core needle biopsy (CNB) can be performed under ultrasound guidance. However, the disadvantages of CNB are it requires local anesthesia, it is more painful, and it has a higher risk of facial nerve injuries and hematoma [16].

## Conclusions

Intraparotid facial nerve schwannomas are difficult to be diagnosed preoperatively, especially when the typical MRI features of target sign on T2WI, growth toward the facial canal, and string sign are not demonstrated. The majority of PFNS are diagnosed intraoperatively or postoperatively. Surgery with acceptable neurologic function is the mainstay of treatment.
